# *TP53* rs1042522 C>G polymorphism and Wilms tumor susceptibility in Chinese children: a four-center case–control study

**DOI:** 10.1042/BSR20181891

**Published:** 2019-01-22

**Authors:** Peng Liu, Zhenjian Zhuo, Wenya Li, Jiwen Cheng, Haixia Zhou, Jing He, Jiao Zhang, Jiaxiang Wang

**Affiliations:** 1 Department of Pediatric Intensive Care Unit, the First Affiliated Hospital of Zhengzhou University, Zhengzhou 450052, Henan, China; 2 School of Chinese Medicine, Faculty of Medicine, The Chinese University of Hong Kong, Hong Kong 999077, China; 3 Department of Pediatric Surgery, the First Affiliated Hospital of Zhengzhou University, Zhengzhou 450052, Henan, China; 4 Department of Pediatric Surgery, the Second Affiliated Hospital of Xi’an Jiaotong University, Xi’an 710004, Shaanxi, China; 5 Department of Hematology, The Second Affiliated Hospital and Yuying Children’s Hospital of Wenzhou Medical University, Wenzhou 325027, Zhejiang, China; 6 Department of Pediatric Surgery, Guangzhou Institute of Pediatrics, Guangzhou Women and Children’s Medical Center, Guangzhou Medical University, Guangzhou 510623, Guangdong, China

**Keywords:** polymorphism, rs1042522 C>G, susceptibility, TP53, Wilms tumor

## Abstract

Wilms tumor is the most common renal malignancy that occurs in children. *TP53* gene is considered as a tumor-suppressing gene through controlling cell growth. *TP53* gene rs1042522 C>G (Arg72Pro) polymorphism is widely investigated in various types of cancers. However, it is not established if *TP53* rs1042522 C>G polymorphism is a candidate variant for Wilms tumor risk. The aim of the study was to determine whether *TP53* rs1042522 C>G polymorphism is responsible for the risk of Wilms tumor in Chinese children. All subjects (355 cases and 1070 controls) from four centers of China were genotyped for rs1042522 C>G polymorphism. The effect of rs1042522 C>G polymorphism on Wilms tumor prevalence was analyzed using logistic regression models. We failed to detect a significant relationship between rs1042522 C>G polymorphism and Wilms tumor risk. Further stratification analysis also could not detect a significant relationship. We conclude that *TP53* rs1042522 C>G polymorphism might not have enough impact on the risk of Wilms tumor. More validation study with larger sample size will be required to better define the role of *TP53* rs1042522 C>G polymorphism in Wilms tumor risk.

## Introduction

Wilms tumor (nephroblastoma) is a most frequently occurring renal malignancy in children, with a prevalence of nearly 1 in 10,000 in Western countries [1–3]. In China, the incidence rate of Wilms tumor is approximately 3.3 in one million children between 2002 and 2010 [4]. Wilms tumor mirrors the different stages of kidney development and commonly presents epithelial, stromal, and blastemal (undifferentiated) components in varying proportions [5]. Wilms tumor typically develops within precursor lesions known as intralobar nephrogenic rests (ILNRs) and perilobar nephrogenic rests (PLNRs) [6]. The overall 5-year survival rate exceeds the benchmark of 90% in developed countries [7–9]. However, nearly 25% of Wilms tumor patients relapse [10,11]. Moreover, disappointing events such as 25% of survivors with severe chronic health conditions and high treatment cost await to be solved.

Nearly 98% of Wilms tumors are sporadic, whereas the rest 2% of cases have at least one relatives suffering from Wilms tumor [12]. Up to now, the major genetic knowledge of Wilms tumor was largely restricted to mutations of Wilms tumor gene 1 (*WT1*), Wilms tumor gene on the X chromosome (*WTX*), tumor protein 53 (*TP53*), catenin β 1 (*CTNNB1*), and the imprinted 11p15 region [13–15]. In addition, many novel mutations in different genes were also revealed by genomic analyses [16–20]. However, all the identified genetic variants still could not explicitly elucidate the carcinogenesis of Wilms tumor. Therefore, the present study aimed to identifying more genetic causes of Wilms tumor.

*TP53* gene, a highly polymorphic gene in human cancers, is located on chromosome 17p13.1 [21]. Its encoded protein, p53 protein, is involved in various cellular activities such as cell cycle control, senescence, apoptosis, and maintenance of DNA integrity [22–26]. Evidence are emerging about the genetic impact of *TP53* gene on the risk of various cancers [27]. *TP53* rs1042522 C>G polymorphism ranks the most widely investigated polymorphism. However, there lacks multicenter study on the association between *TP53* rs1042522 C>G polymorphism and Wilms tumor risk, except our recent investigation using relatively small sample size [28]. Considering the critical role of *TP53* rs1042522 C>G polymorphism in cancer risk, we selected this polymorphism for further analyzation. The main objective of the present study was to analyze the relationship between *TP53* rs1042522 C>G polymorphism and Wilms tumor risk in a much larger sample size.

## Materials and methods

### Study subjects

A total of 355 cases from four hospitals (Guangzhou Women and Children’s Medical Center, The First Affiliated Hospital of Zhengzhou University, The Second Affiliated Hospital and Yuying Children’s Hospital of Wenzhou Medical University, and Second Affiliated Hospital of Xi’an Jiao Tong University) located in four different cities of China (Guangzhou, Zhengzhou, Wenzhou, and Xi’an) participated in this project. The cases were individuals diagnosed with Wilms tumor. A total of 1070 controls were enrolled randomly from those who resided in the same areas of cases at the same time [29–34]. Recruitment details of the subjects were described as previously [28,35–37]. Trained medical staff collected patients’ information on age, gender, and clinical stages. Personal written informed consent was collected prior study. The present study was approved by all the four Institutional Review Boards of the respective hospitals.

### Genotyping

All subjects donated a blood sample at recruitment. DNA was then extracted from the blood sample using a TIANamp Blood DNA Kit (TianGen Biotech Co. Ltd., Beijing, China). Genotyping was conducted using TaqMan methodology as previously described [38–41]. Negative controls with water were also included to ensure genotyping accuracy. We reran 10% of samples in duplicate to test reproducibility.

### Statistical analysis

The χ^2^ test for genotype distribution was applied to detect deviation from the Hardy–Weinberg equilibrium (HWE) in control group. Two-sided χ^2^ test was also performed in a case–control analysis to analyze the difference of clinical-pathologic characteristics. Wilms tumor risk was estimated as odds ratios (ORs) and 95% confidence intervals (CIs), based on unconditional logistic regression adjusted for age and gender. *P*<0.05 was considered as statistical significant. SAS statistical package, version 9.1 (SAS Institute, Cary, NC) was used to conduct the analyses.

## Results

### Population characters

The frequency distribution of selected variables of all the subjects was presented in Table 1. No significant differences of age and gender were detected between cases and controls (*P*=0.131 for age and *P*=0.182 for gender). In classified clinical stages of the cases, 33.52% were diagnosed as clinical stages I, 25.92% were as II, 22.25% were as III, 13.24% were as IV, and 5.07% were as not available (NA).

**Table 1:** Frequency distribution of selected variables in Wilms tumor patients and controls

Variables	Cases (*n*=355)		Controls (*n*=1070)		P*
	No.	%	No.	%	
Age range, month	1–148.63		0.03–156		0.131
Mean ± SD	30.67 ± 23.96		32.27 ± 26.89		
≤18	125	35.21	425	39.72	
>18	230	64.79	645	60.28	
Gender					0.182
Female	163	45.92	448	41.87	
Male	192	54.08	622	58.13	
Clinical stages					
I	119	33.52			
II	92	25.92			
III	79	22.25			
IV	47	13.24			
NA	18	5.07			

Abbreviations: SD, standard deviation; NA, not available.

* Two-sided *χ^2^* test for distributions between Wilms tumor patients and cancer-free controls.

### *TP53* gene rs1042522 C>G polymorphism and Wilms tumor susceptibility

A total of 354 patients and 1069 healthy controls were successfully genotyped. The association between *TP53* rs1042522 C>G polymorphism and Wilms risk was listed in Table 2. The controls were in HWE for rs1042522 C>G polymorphism (HWE = 0.331). Genotype frequencies of CC, CG, and GG genotypes among cases were 27.40%, 52.54%, and 20.06% while in controls 28.34%, 51.17%, and 20.49%, respectively. No statistically significant relationship was observed between *TP53* rs1042522 C>G polymorphism and Wilms tumor risk, either adjustment for age and gender or not.

**Table 2:** Association between *TP53* rs1042522 C>G polymorphism and Wilms tumor risk

Genotype	Cases (*N*=354)	Controls (*N*=1069)	P*	Crude OR (95% CI)	*P*	Adjusted OR (95% CI)^†^	*P*^†^
rs1042522 (HWE = 0.331)							
CC	97 (27.40)	303 (28.34)		1.00		1.00	
CG	186 (52.54)	547 (51.17)		1.06 (0.80–1.41)	0.676	1.06 (0.80–1.41)	0.680
GG	71 (20.06)	219 (20.49)		1.01 (0.71–1.44)	0.944	1.01 (0.71–1.44)	0.939
Additive			0.902	1.01 (0.85–1.20)	0.904	1.01 (0.85–1.20)	0.900
Dominant	257 (72.60)	766 (71.66)	0.732	1.05 (0.80–1.37)	0.734	1.05 (0.80–1.37)	0.733
Recessive	283 (79.94)	850 (79.51)	0.862	0.97 (0.72–1.31)	0.862	0.98 (0.72–1.32)	0.870

Abbreviations: CI, confidence interval; HWE, Hardy–Weinberg equilibrium; OR, odds ratio.

* *χ^2^* test for genotype distributions between Wilms tumor patients and cancer-free controls.

† Adjusted for age and gender.

### Stratification analysis

To deeply determine whether rs1042522 C>G polymorphism influences Wilms tumor risk under certain strata, we carried out stratification analysis by age, gender, and clinical stages. Yet, we still failed to detect any positive relationship of interest among all the analyzed strata (Table 3).

**Table 3:** Stratification analysis of TP53 rs1042522 C>G polymorphism with Wilms tumor risk

Variables	rs1042522 (Cases/Controls)		Crude OR (95% CI)	*P*	Adjusted OR* (95% CI)	P*
	CC	CG/GG				
Age, month						
≤18	35/119	90/306	1.00 (0.64–1.56)	1.000	1.00 (0.64–1.55)	0.981
>18	62/184	167/460	1.08 (0.77–1.51)	0.665	1.07 (0.76–1.50)	0.699
Gender						
Females	47/124	116/323	0.95 (0.64–1.41)	0.790	0.95 (0.64–-1.41)	0.793
Males	50/179	141/443	1.14 (0.79–1.64)	0.485	1.14 (0.79–1.64)	0.497
Clinical stages						
I	39/303	80/766	0.81 (0.54–1.22)	0.312	0.81 (0.54–1.22)	0.317
II	22/303	69/766	1.24 (0.75–2.04)	0.396	1.24 (0.76–2.05)	0.392
III	17/303	62/766	1.44 (0.83–2.51)	0.194	1.44 (0.83–2.51)	0.196
IV	17/303	30/766	0.70 (0.38–1.28)	0.248	0.70 (0.38–1.28)	0.243
I+II	61/303	149/766	0.97 (0.70–1.34)	0.836	0.97 (0.70–1.34)	0.847
III+IV	34/303	92/766	1.07 (0.71–1.62)	0.748	1.07 (0.70–1.62)	0.756

OR, odds ratio; CI, confidence interval.

* Adjusted for age and gender, omitting the corresponding variable.

## Discussion

Here, in order to validate the role of *TP53* rs1042522 C>G polymorphism on Wilms tumor risk, we conducted a four-center case–control study with 355 cases and 1070 controls in Chinese population. To our knowledge, this is by now the largest case–control study regarding the relationship between *TP53* rs1042522 C>G polymorphism and Wilms tumor risk.

*TP53*, a tumor suppressor gene, is located on chromosome 17p13 and is 20 kb long in humans [42]. Hundreds of polymorphisms in *TP53* gene have been identified to be associated with cancer risk [43]. Among them, *TP53* rs1042522 C>G polymorphism ranks the most widely investigated polymorphism. The rs1042522 C>G polymorphism at codon 72 in exon 4 of the *TP53* gene is the non-synonymous substitution of C>G, which results in an amino acid transversion from arginine (Arg) to proline (Pro) [44]. Such amino acid transversion alters the primary structures and consequentially biochemical functions. Experimental studies revealed that *TP53* codon 72 Pro variant was related to a reduction in inducing apoptosis, while an increase in inducing cell cycle arrest [45,46].

Given the critical role of *TP53* rs1042522 C>G polymorphism in carcinogenesis, many researchers attempted to elucidate their roles in certain cancer type risk. A previous study in an Indian population explored the association between *TP53* gene rs1042522 C>G polymorphism and the risk of thyroid cancer. The authors found that rs1042522 C>G polymorphism conferred higher susceptibility to thyroid cancer [47]. Chen et al. [48] genotyped 168 retinoblastoma patients and 185 adult controls from China. They observed no significant difference in allele or genotypic frequencies of *TP53* gene rs1042522 C>G polymorphism between cases and controls. By genotyping 288 healthy subjects and 286 neuroblastoma patients in European descent, Cattelani et al. [49] found that the *TP53* gene rs1042522 C>G polymorphism had no significant impact on conferring to risk of neuroblastoma. For breast cancer, the role of *TP53* gene rs1042522 C>G polymorphism vary from protective [50] to no association [51] or increase [52] in risk. The discrepancies of the above-mentioned conclusions may be attributed to the differences in cancer types, population sources, geographical environment, and sample sizes. Therefore, elucidating the exact role of *TP53* rs1042522 C>G polymorphism on certain type of cancer under certain population is of great necessity.

*TP53* rs1042522 C>G polymorphism in influencing Wilms tumor risk remains largely unknown. Andrade et al. [53] was the first to assess the impact of *TP53* polymorphisms on risk of Wilms tumor. They found the rs1042522 C allele carriers may be associated with increased Wilms tumor risk, using 46 cases and 300 controls from Brazil. In our previous experiments, we failed to obtain evidence that *TP53* rs1042522 C>G polymorphism predisposes to Wilms tumor risk [28]. Yet this previous study only included 145 Wilms tumor cases and 531 controls enrolled from one single hospital. To establish a more precise relationship between *TP53* rs1042522 C>G polymorphism and Wilms tumor risk, we enlarge our sample size, with 355 cases and 1070 controls from four hospitals located in four different cities of China. Unexpectedly, we still failed to detect any significant contribution of the *TP53* rs1042522 C>G polymorphism to Wilms tumor risk. Plausible interpretations of the null association include relatively small sample size and low-penetrance susceptibility of single polymorphism. We speculated that differences in the study population created a disparity between the study conducted by Andrade et al. [53] and our study.

Several limitations accompany the current article. The moderate sample size limited the statistical power to detect weak interactions. Some findings might be just due to chance, particularly for stratification analysis. All the samples were selected from Chinese Han population, and that the lack of crossing ethnicity data is just another one of the limitation of the present study. Furthermore, we only concentrated on genetic factors; analysis of environmental factors and genetic–environmental factors were not conducted. Last, the only one analyzed polymorphism here is far from enough to fully elucidate the risk of Wilms tumor.

Notwithstanding these limitations, our data provided a relative strong evidence that single *TP53* gene rs1042522 C>G polymorphism may not have enough impact on the risk of Wilms tumor. Further studies into the molecular mechanisms of *TP53* gene rs1042522 C>G polymorphism may provide deeper insights into the etiology of Wilms tumor.

## Abbreviations

Arg: arginine
CI: confidence interval
*CTNNB1*: catenin β 1
HWE: Hardy–Weinberg equilibrium
ILNR: intralobar nephrogenic rest
NA: not available
OR: odds ratio
PLNR: perilobar nephrogenic rest
Pro: proline
*TP53*: tumor protein 53
*WT1*: Wilms tumor gene 1
*WTX*: Wilms tumor gene on the X chromosome
